# A new analytical framework of 'continuum of prevention and care' to maximize HIV case detection and retention in care in Vietnam

**DOI:** 10.1186/1472-6963-12-483

**Published:** 2012-12-29

**Authors:** Masami Fujita, Krishna C Poudel, Nhan Do Thi, Duong Bui Duc, Kinh Nguyen Van, Kimberly Green, Thu Nguyen Thi Minh, Masaya Kato, David Jacka, Thuy Cao Thi Thanh, Long Nguyen Thanh, Masamine Jimba

**Affiliations:** 1 World Health Organization Cambodia Office, No. 177-179 Pasteur (St.51) (corner 254) Sangkat Chak Tomouk, P.O. Box 1217, , Phnom Penh, Cambodia; 2 Department of Public Health, School of Public Health and Health Sciences, University of Massachusetts Amherst, 316 Arnold House, 715 North Pleasant St, Amherst, MA, 01003-9304, USA; 3 Vietnam Authority of HIV/AIDS Control, Ministry of Health Vietnam, 135/3 Nui Truc, Ba Dinh, Hanoi, Vietnam; 4 National Hospital of Tropical Diseases, 78 Giai Phong Street, Hanoi, Vietnam; 5 FHI 360, Demmco House, 1st Dzorwulu Crescent, Accra, Ghana; 6 World Health Organization Vietnam Office, 63 Tran Hung Dao, Hoan Kiem District, P.O. Box 52, Hanoi, Vietnam; 7 Department of Community and Global Health, Graduate School of Medicine, The University of Tokyo, 7-3-1 Hongo, Bunkyo-ku, Tokyo, Japan

**Keywords:** HIV, Health services, Health care systems, Developing countries

## Abstract

**Background:**

The global initiative ‘Treatment 2.0’ calls for expanding the evidence base of optimal HIV service delivery models to maximize HIV case detection and retention in care. However limited systematic assessment has been conducted in countries with concentrated HIV epidemic. We aimed to assess HIV service availability and service connectedness in Vietnam.

**Methods:**

We developed a new analytical framework of the continuum of prevention and care (COPC). Using the framework, we examined HIV service delivery in Vietnam. Specifically, we analyzed HIV service availability including geographical distribution and decentralization and service connectedness across multiple services and dimensions. We then identified system-related strengths and constraints in improving HIV case detection and retention in care. This was accomplished by reviewing related published and unpublished documents including existing service delivery data.

**Results:**

Identified strengths included: decentralized HIV outpatient clinics that offer comprehensive care at the district level particularly in high HIV burden provinces; functional chronic care management for antiretroviral treatment (ART) with the involvement of people living with HIV and the links to community- and home-based care; HIV testing and counseling integrated into tuberculosis and antenatal care services in districts supported by donor-funded projects, and extensive peer outreach networks that reduce barriers for the most-at-risk populations to access services. Constraints included: fragmented local coordination mechanisms for HIV-related health services; lack of systems to monitor the expansion of HIV outpatient clinics that offer comprehensive care; underdevelopment of pre-ART care; insufficient linkage from HIV testing and counseling to pre-ART care; inadequate access to HIV-related services in districts not supported by donor-funded projects particularly in middle and low burden provinces and in mountainous remote areas; and no systematic monitoring of referral services.

**Conclusions:**

Our COPC analytical framework was instrumental in identifying system-related strengths and constraints that contribute to HIV case detection and retention in care. The national HIV program plans to strengthen provincial programming by re-defining various service linkages and accelerate the transition from project-based approach to integrated service delivery in line with the ‘Treatment 2.0’ initiative.

## Background

Many resource-limited countries are facing significant challenges to delivering HIV health services. The first challenge is delayed HIV diagnosis and late presentation for antiretroviral treatment (ART) resulting in elevated morbidity and mortality [1] and in diminished benefit of ART as prevention [2]. The second one is a slow progress in expanding prevention of mother-to-child transmission (PMTCT) and tuberculosis (TB) - HIV collaborative activities [3]. Third, HIV care and support services are under-prioritized and under-funded, resulting in reduced access for pre-ART care, ART and end-of life patients [4]. Fourth, increasing number of PLHIV on ART for many years are facing long-term ART side effects, drug resistance, co-morbidities, and psycho-social constraints [5,6]. In addition to these challenges, the needs of most-at-risk populations (MARPs) have been overlooked in terms of HIV prevention, testing and counseling, and treatment particularly in Asia [7].

In these poor countries, project-based service delivery systems have been developed through urgent service expansion with massive donor funding over the last decade. In light of the current economic downturn, the new global initiative ‘Treatment 2.0’ highlights the needs to adapt delivery systems by decentralizing and integrating HIV treatment with other services and engaging community [8]. These services include drug dependence service, maternal and child health (MCH) and tuberculosis (TB) services. The adaptation of delivery systems is expected to maximize HIV case detection and retention in care. The initiative calls for expanding the evidence base on optimal service delivery models in a variety of settings [9].

To date, limited systematic assessment has been conducted on overall models of HIV service delivery. Analyses of HIV service delivery have been fragmented. The focus has been on either one component of HIV health services such as ART [10], or integration between two services such as HIV and TB [11], HIV and MCH [12] or HIV and family planning [13]. The concept of the continuum of care, more recently understood as the continuum of prevention and care (COPC), has been used to coordinate and link the health facilities, the community and other sectors under one coherent framework [14-18]. We theorized that assessing service delivery models using the COPC concept could contribute to the optimization of HIV service delivery particularly to improving HIV case detection and retention in care in concentrated epidemic settings.

The first assessment using the COPC framework was conducted in Vietnam among other Asian countries. The HIV epidemic in Vietnam is largely concentrated among injecting drug users (IDUs), sex workers (SWs) and men who have sex with men (MSM). Adult HIV prevalence (aged 15–49) was estimated to be 0.44% with an estimated 254,000 PLHIV in 2010 [19]. HIV cases have been reported nationwide in all 63 provinces/cities, 98% of districts, and 74% of wards/communes (VAAC: Unpublished report; 2010).

This study aimed to assess HIV service delivery in Vietnam by identifying system related strengths and constraints that are common to multiple elements of HIV health services using the lens of the COPC. Specifically, we aimed to assess service availability including geographical distribution and decentralization and service connectedness across multiple services and dimensions that contribute to HIV case detection and retention in care. Vietnam has several relevant characteristics in common with many other Asian countries, such as low HIV prevalence in the general population but high prevalence among various MARPs [7], enormous variation of HIV prevalence across geographical areas, and structural barriers related to working with MARPs [20]. By providing a systematic analysis of the experience in Vietnam, we hope that such information will be useful in strengthening HIV service delivery of other resource-limited countries, particularly in Asia.

## Methods

### Definition of COPC

The COPC originates from the concept of the continuum of care developed in the 1970s to offer continuity of care for the elderly [21]. This concept emerged in the field of HIV/AIDS in 1990s when there were limited measures to prevent and treat HIV [22,23]. As the availability of HIV prevention, care and treatment has improved in resource limited settings, the concept has further evolved to include prevention [24,25].

The COPC can be defined as a coordinated network of prevention, treatment, care, and support activities. This network includes government, community-based organizations, non-government organizations, PLHIV and/or MARPs, their family members and others. This network spans different levels of the health system including the community. The resulting activities provide comprehensive services for adults, children, youth and families vulnerable to, living with and affected by HIV over the long-term [26] (Fujita M and Green K: Unpublished presentation; 2010).

A critical element of the COPC is to establish a comprehensive care site (CCS) as a central mechanism of a local service network. The CCS offers not only clinical care but a wide range of associated services. Such services include health education, psychosocial support, links to other services and community- and home-based care (CHBC), as well as opportunities for the involvement of affected communities such as MARPs and PLHIV. The names given to the CCS differ across Asia, such as the Day Care Centre, the Comprehensive Continuum of Care Centre, and the Friend-Help-Friend Centre [27]. Since HIV outpatient clinic is a common term used for HIV care and treatment facilities in Vietnam, in this paper, the term HIV outpatient ‘plus’ is used to denote activities and services of the CCS.

### Analytical framework

**Figure 1:**
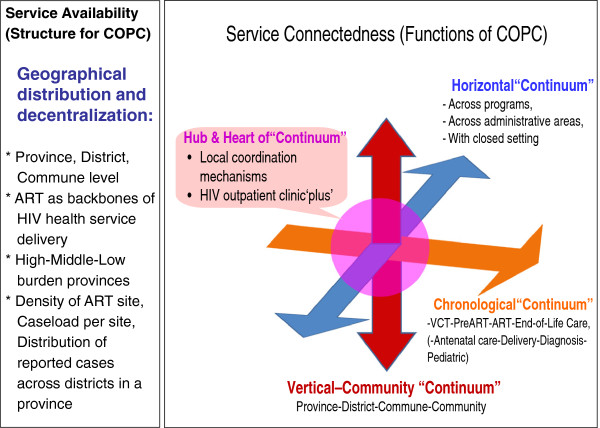
Framework for assessing HIV health services from the viewpoint of COPC.

We developed a new analytical framework of COPC for the review of HIV service delivery in Vietnam (Figure 1). The COPC concept can be applied to analyze HIV service delivery across several dimensions: (i) geographical distribution of MARPs and PLHIV; (ii) availability of HIV health services including their geographical distribution and decentralization; (iii) connectedness between different elements of HIV health services; (iv) outcomes and impact of service availability and connectedness at a population level; and (v) interaction between HIV service delivery and other parts of the health system. To meet our study objectives and the availability of existing data, we focused primarily on (ii) and (iii) above.

The *service availability* assessment focused on examining geographical distribution of services and service delivery decentralization. In assessing *service connectedness,* we defined four functions: 1) local coordination mechanisms and HIV outpatient clinic ‘plus’ (Hub and Heart of Continuum); 2) chronic care provision including self-care, peer support and patient follow-up and tracking as well as recording systems throughout the stages of HIV diagnosis, pre-ART care, ART and end-of-life care (Chronological Continuum); 3) linkages and/or integration across HIV and other health services, across different geographical administrative areas, and across community health facilities and closed settings such as detention centers for drug users and SWs and prisons (Horizontal Continuum); and 4) service linkages across commune, district and provincial levels including prevention outreach and CHBC (Vertical-Community Continuum).

### Literature search and review

We conducted extensive review of HIV program and project reports, policy documents, legislation, and published articles. A primary source was the office of the Vietnam Authority (formerly ‘Vietnam Administration’) of HIV/AIDS Control (VAAC), Ministry of Health. We also searched electronic databases such as Popline, Web of Science, Medline, Embase, and Google scholar. Using these sources, we identified articles published in English between January 1990 and August 2011. Keywords used for the search include HIV, AIDS, prevention, treatment, care, support and Vietnam in appropriate combinations and syntax according to the database. To fully explore the situation in Vietnam to the end of 2010, we also explored the reference lists of the key identified papers and consulted program staff of various organizations. We then analyzed the data obtained using the COPC analytical framework and identified strengths and constraints of HIV health service delivery in improving HIV case detection and retention in care.

## Results

### Service availability including geographical distribution and decentralization

Organization of local health facilities follows the administrative divisions of 63 provinces, 697 districts and 11,112 communes [28]. TB diagnosis (smear) and delivery services were noted to be available in all districts. All communes were charged with providing TB treatment and antenatal care services.

HIV prevention for MARPs and HIV care and treatment has been rapidly expanded since 2004. Evolution of HIV health services are summarized in Table 1[29-32]. By 2010 HIV prevention targeting MARPs included needle and syringe programs for IDUs. These programs were implemented in 3,333 communes in 352 districts or 51% of all districts in 43 provinces mainly by 1,792 peer educators (Table 2) (VAAC: Unpublished report; 2010). Condom use program targeting SWs involved 3,123 peer educators. In addition, voluntary counseling and testing (VCT) and ART services co-existed in the same districts in most cases. That is, 175 districts had VCT and 167 of these districts had ART sites. At the provincial level, both VCT and ART were present in all provinces. PMTCT services involving antiretroviral prophylaxis were available in 133 districts in 63 provinces. Methadone maintenance therapy (MMT) was available in 11 districts in 4 provinces.

**Table 1:** Evolution of HIV health services in Vietnam

**Year**	**Evolution**
Early 1990s	Small-scale responses initiated at the local level such as local government led needle and syringe programs and peer support activities.
Mid 1990s	The Ministry of Health initiated a HIV prevention campaign followed by commune health station based basic care and support for PLHIV in three provinces.
Early 2000s	The 100% condom use program piloted by the Ministry of Health and a number of needle and syringe programs implemented by non-governmental organizations.
	Establishment of HIV clinical services at national hospitals followed by district level HIV outpatient clinics offering comprehensive care in Ho Chi Minh City.
2004 (and onward)	Health sector-led large scale expansion of HIV prevention, care and treatment initiated (supported by the United States, the United Kingdom, the World Bank and the Global Fund)
	For HIV care and treatment, HIV outpatient clinics expanded based on the experiences of a number of model sites.
	Community- and home-based care (CHBC) expanded in different forms, such as (i) HIV outpatient clinic based; (ii) Stand-alone model run by PLHIV groups, faith-based organizations or local non-governmental organizations; (iii) Led by Women’s Union; and (iv) Commune health station based.
	ART expanded in administrative detention centers for IDUs and SWs, followed by in prisons.
2007	National Plan of Action on Harm Reduction approved. It stipulated that HIV officers at provincial and district health services play a central role in mobilizing peer educators from current or former IDUs/SWs and entertainment establishment owners/managers.
2008	Under the legal framework of the Law on HIV and its decree, the national pilot Methadone Maintenance Therapy (MMT) program began in two provinces,

(Source: [29-32], Le TG: Unpublished presentation; 2005, Fujita M and Green K: Unpublished presentation; 2010, VAAC: Unpublished report, 2010).

**Table 2:** Strengths and constraints of HIV health service delivery in improving HIV case detection and retention in care

	**Strengths**	**Constraints**
**Availability**	- Outreach peer educators in more than half of districts [D,R]	- Less than one-third of districts offering VCT/ART at district level in middle/low HIV burden provinces [D,R]
	- Two-thirds of districts offering VCT/ART at district level in high HIV burden provinces [D,R]	- Lack of physically accessible VCT/ART in remote areas in high/middle burden provinces [D,R]
**Connectedness**		
**Hub & Heart**	- Coordination mechanism between administrative detention centers and HIV outpatient clinics emerging [R]	- No coordination mechanism between districts with VCT/ART and those without [D,R]
	- HIV outpatient clinic ‘plus’ at district level expanded in high and middle burden provinces [D,R]	- Clinical services only in government funded HIV outpatient clinic at provincial level [D,R]
		- No system to monitor expansion of outpatient clinic ‘plus’ [D,R]
**Chronological**	- Chronic care based ART case management established for IDU and non-IDU [R]	- Limited capacity to address the needs of PLHIV on ART for many years [R]
	- Palliative care initiated integrated with cancer care [R]	- Pre-ART care under-developed [R]
		- Linkage from VCT to pre-ART care under-developed [R]
**Horizontal**	- HIV testing and counseling integrated into TB and antenatal care in donor funded districts with ART/VCT in high (and middle) burden provinces [D]	- Lack of linkage for HIV-TB and HIV-MCH in non-donor funded districts without VCT/ART in middle/low burden provinces [D]
	- Referral system between administrative detention centers and HIV outpatient clinics being developed [R]	- HIV service register not designed to facilitate TB/HIV and PMTCT [R]
**Vertical**	- Extensive mobilization of peer educators to facilitate MARPs to access VCT [D]	- Access to HIV testing and care and treatment in advanced stage of HIV infection [D,R]
	- Alternative approaches to reach hidden MARPs emerging [D]	- Health workers commonly providing verbal advice only to patients for referral across different levels of health facilities [R]
	- CHBC models mobilizing a wide range of stakeholders [R]	- No system to monitor referral services [R]

Remark:

[D] stands for a strength or constraint that is related to HIV case detection.

[R] stands for a strength or constraint that is related to retention in care.

The density of ART sites in the provinces (number of ART sites / number of districts × 100) was 66% in the high, 29% in the middle, and 15% in the low burden provinces (Table 3). It was noted that a number of high and middle burden provinces had mountainous remote areas where a substantive portion of MARPs and PLHIV had limited physical access to health facilities.

**Table 3:** Availability of ART sites according to different levels of HIV burden in 2009

	**High burden province**		**Middle burden province**		**Low burden province**	
	**n**	**(%)**	**n**	**(%)**	**n**	**(%)**
Number of provinces	8		29		26	
Number of districts	122		300		268	
Number of ART sites	80		88		39	
District with ART	65		72		30	
Density of ART site (%)		65.6		29.3		14.6
(Number of ART sites / Number of districts x 100)						
Estimated number of patients needing ART	36,682	(58.0)	21,197	(33.5)	5,409	(8.5)
Number of patients on ART	25,449	(70.7)	8,464	(23.5)	2,095	(5.8)
Estimated number of patients needing ART per district	301		71		20	
Number of ART patients per district	209		28		8	
ART coverage (%)						
(Number of patients on ART / Estimated number of patients needing ART x 100)		69.4		39.9		38.7

Remark: High burden province: over 300 reported cases per district and over 150 patients needing ART per district;

Middle burden province: over 100 reported cases per district or over 50 patients needing ART per district;

Low burden: less than 100 reported cases per district and less than 50 patients needing ART per district.

(Source: COPC review group: Unpublished report; 2010, Do TN: Unpublished presentation; 2010).

### Service connectedness

1) Hub and heart of continuum

a) Local coordination mechanisms

Mechanisms for local coordination of HIV and other related health services were stipulated in several official documents from the Ministry of Health and its donor-funded projects. These documents included national guidance on HIV care and treatment, TB/HIV, PMTCT as well as project guidance on HIV prevention for MARPs [33-39]. Consequently, a variety of coordination committees for HIV-related services were formed in some areas, while none were formed in other areas. Most provinces lacked coordination mechanisms for HIV-related services between districts. That is, districts providing ART/VCT services rarely coordinated with districts without such services (Bui DD: Unpublished presentation; 2010).

b) HIV outpatient clinic ‘plus’

The national guidance on HIV care and treatment stipulated the responsibilities undertaken in the HIV outpatient clinics [33,34]. These stipulations included the provision of clinical services, health education, prevention services, and psychosocial support. Other stipulations included the provision of linkages with other relevant health services and the involvement of PLHIV as members of care teams and local HIV treatment committees.

Large donor funded initiatives supported expansion of HIV outpatient clinics mainly in high and middle burden provinces (Table 2). In addition to the clinical services, several functions were added consistent to support the concept of the HIV outpatient clinic ‘plus’. These functions included: (i) facilitating HIV positive MARPs to access HIV outpatient clinics; (ii) involving HIV positive MARPs in HIV prevention initiatives targeting MARPs; (iii) supporting treatment adherence in collaboration with commune health stations, peer educators and CHBC teams; (iv) linking with TB and MCH services as well as closed settings; (v) establishing patient referral procedures to specialized hospitals [38-40]. Despite these initiatives, however, some HIV outpatient clinics were known to be only providing clinical services, especially in low burden provinces where limited donor funded projects were operating.

2) Chronological continuum

National guidance documents stipulated case management procedures and provided standardized longitudinal registers for pre-ART care and ART [41]. These documents were in line with the chronic care principles including self-care, peer support, and patient follow-up information systems [42].

**Figure 2:**
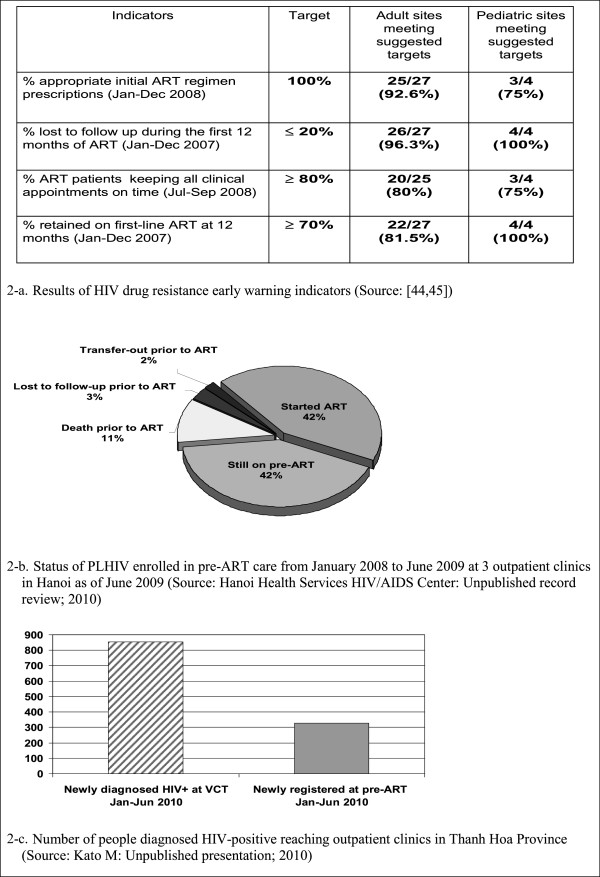
Outcomes of people diagnosed HIV-positive and initiated ART.

HIV outpatient clinics tended to actively prepare and track patients for ART by mobilizing PLHIV peer support and CHBC. Program data indicated the percentage of adults and children with HIV still alive and known to be on treatment 12 months after initiation of ART was 84.2% [43]. HIV drug resistance early warning indicators indicated good adherence to appointment schedule and low level of lost-to-follow-up despite a large proportion of the patients being IDUs (Figure 2-a) [44,45]. Furthermore, a study conducted a 2-year prospective cohort analysis of patients taking ART in two HIV outpatient clinic ‘plus’ sites in Ho Chi Minh City. It revealed the change of median CD4 count over the 24-month follow-up period among patients who ever injected illicit opiates was similar to that for those who reported never having injected [46]. In another study in Hanoi [47] viral suppression was not statistically different among the patients who used drug in the previous six months versus those who did not use it after at least six months of ART initiation.

To meet palliative care needs [48], the Ministry of Health [49] developed the guidelines on palliative care for cancer and AIDS patients in 2006. The ministry also improved opioid prescribing regulations in 2008 and trained over 400 physicians by early 2010 [50]. As a result, palliative care services have started in both hospitals and communities [51].

Patient follow-up and tracking for pre-ART care appeared to be considerably less operational than for ART. Results from an ad hoc assessment indicated that a substantial number of PLHIV might be dying or lost-to-follow-up during the pre-ART period (Figure 2-b) (Hanoi Health Services HIV/AIDS Center: Unpublished record review; 2010). In 2009, monitoring of attrition from pre-ART care started as part of HIV drug resistance early warning indicators monitoring [52].

Existing referral forms were not used extensively to facilitate the referral process from VCT to pre-ART care. An ad hoc assessment in one province indicated a large gap between the number of people diagnosed as HIV positive at VCT and the number of people enrolled in pre-ART care (Figure 2-c) (Kato M: Unpublished presentation; 2010). No specific national guidance or patient tracking system was established for this process.

3) Horizontal continuum

Collaborative activities between HIV and TB services and HIV and MCH services (PMTCT) were expanded mainly through donor funded projects. These collaborative efforts were consistent with the national guidance developed by concerned national programs [35,36] (Table 2).

However, these projects tended to support specific districts rather than a provincial network. In districts without donor-funded VCT/ART, scarcely any health workers were charged with providing HIV health services (COPC review group: Unpublished report, 2010). In these districts, TB and antenatal care services were rarely equipped to implement provider-initiated testing and counseling for TB cases and for pregnant women. As a result, in 2009 the percentage of estimated HIV-positive incident TB cases that received treatment for TB and HIV was as low as 27.5% [43]. Similarly, the percentage of HIV-positive pregnant women who received antiretroviral medicines to reduce the risk of mother-to-child transmission was 32.3%.

Standard TB registers included a section for recording HIV status. However, antenatal care registers did not include a space to record HIV status among pregnant women. Pre-ART and ART registers did not have a section for recording TB diagnosis and treatment or for pregnancy status. Patient-held records provided this additional information but were used in only a limited number of sites.

All the methadone maintenance therapy (MMT) sites were located in districts where VCT/ART was present [53] (VAAC: Unpublished report; 2010). Some of the current MMT sites were stand-alone and physically separate from HIV outpatient clinics while others were co-located with HIV outpatient clinics. Efforts were made to strengthen linkages between MMT and other HIV health services as part of the MMT scale-up.

4) Vertical continuum

a) Linkages across different levels of health services

PLHIV suspected of active TB were often required to travel long distances across the community, district, and provincial levels. Diagnosis of smear-negative and extra-pulmonary TB was mostly performed at the provincial level. Similarly, a number of PMTCT services were provided mainly at the provincial level. These services included planned delivery of HIV positive women, early infant diagnosis and pediatric treatment. In addition, the provincial level was responsible for confirmation of HIV treatment failure and prescription of second line ART regimens in most provinces [41].

All of these patient flows required robust referral systems including patient information sharing across the different levels. Commonly, a doctor simply instructed a patient to go to another facility often without a referral form (Table 2). There were no routine mechanisms to monitor the functioning of the referral process (COPC review group: Unpublished report; 2010). Respect for administrative boundaries and the hierarchy of authority were reported to often make health workers reluctant to contact their peers in other health facilities.

b) Community response and its linkage with health services

Peer educators for condom use and needle and syringe program were encouraged to systematically refer their clients to VCT (Table 2). An increasing number of MSM peer educators were also recruited particularly in large cities. However, the coverage of HIV prevention programs for male IDUs, female SWs and MSM was only 15.4%, 47.3% and 24%, respectively in 2009 [43]. Similarly, the coverage of HIV testing and counseling among IDUs, female SWs and MSM was low at 17.9%, 34.8% and 19.1%, respectively. Consequently, majority of PLHIV accessed HIV care and treatment services at an advanced stage of HIV infection. Program monitoring data from 2009 indicated 64% of PLHIV started ART at CD4 100/cm^3^ or lower [52].

Alternatives to the peer education approach were reported to be emerging to serve hard-to-reach populations. These alternatives included mobilization of pharmacies, street vendors, self-service boxes and commune health stations for needle and syringe and condom use programs [54]. Civil society partners also began to extend their reach to MSM through internet connections and cruising hot spots.

In most districts, peer educators for prevention and those for care were reported to be managed and supported separately by different donor funded projects. However, there were growing examples of synergy between the activities of the two different peer educator groups [54]. These examples included drop-in-centers for IDUs managed by PLHIV with support from district HIV outpatient clinics, and needle and syringe program activities performed by PLHIV.

## Discussion

### Service availability including geographical distribution and decentralization

Only a quarter of districts had VCT or ART sites, while MARPs had been identified and reached in more than half of the districts. TB diagnosis (smear) and antenatal care services were provided in all the districts throughout the country.

In high burden provinces, estimated ART coverage appeared to be reaching saturation. However, in middle and low burden provinces, the coverage appeared to be relatively low and the ART caseload per facility was too small to warrant further expansion of district ART sites. In some remote areas rated high and middle burden, a substantive number of PLHIV have limited physical access to district health facilities.

To maximize HIV case detection and retention in care, it is therefore crucial to strengthen service connectedness in middle and low burden provinces. Remote areas in high and middle burden provinces require consideration of specific approaches. Such initiatives include further decentralized and/or mobile services [55,56].

### Service connectedness

1) Local coordination mechanisms

Local coordination mechanisms for HIV-related health services vary significantly across different provinces and in some cases they are non-existent. It would be beneficial to review the experiences from different forms of coordination mechanisms across the country to inform future guidance development [40]. In particular, consideration should be given to coordination mechanisms between districts with VCT/ART and those without. One option is to form clusters of districts in each province and to establish clear referral procedures as implemented in a country in Asia [57]. In this way, within the same cluster, districts offering VCT/ART could collaborate with districts not offering VCT/ART services to improve HIV case detection and retention in care.

2) Functions of HIV outpatient clinic

HIV outpatient clinics established multiple functions to the chronological, horizontal and vertical continuum of prevention and care. This was accomplished through mobilizing PLHIV and MARPs peer educators and by developing links to CHBC and other related services as seen in other Asian countries [18,40]. This was particularly evident in high burden provinces.

Early in the epidemic, PLHIV and MARPs were seen as passive recipients of services. Over the past decade, there has been a paradigm shift. When new HIV outpatient clinics were established, health workers encouraged PLHIV and MARPs to work as peer educators, care providers and support-group members. This provided opportunities for health workers to receive feedback on their services. As a result, acceptance and trust of health services among PLHIV and MARPs have improved. This improved relationship facilitated a rapid expansion of peer educators.

However, limited information was available to know the status of more than 160 HIV outpatient clinics across the country. Establishment of a simple system to monitor the functions of these HIV outpatient clinics would help national and local HIV programs to improve HIV case detection and retention in care especially among MARPs.

3) Chronic care

Chronic care systems for PLHIV on ART were well established through peer mobilization, patient follow-up and tracking, and longitudinal monitoring [42,58]. Their effectiveness is reflected in high levels of ART retention and appointment keeping within a twenty-four month period. Such success is evident despite the fact that a large proportion of the patients are IDUs without MMT services [46,47].

However, retention from HIV testing and counseling to pre-ART care and during pre-ART care were sub-optimal as in the case in Sub-Saharan Africa [59]. These processes should be strengthened to accelerate early ART initiation and to introduce ART as prevention among discordant couples [2,60]. It will also improve case management of TB-HIV co-infections and HIV positive pregnant women. Integrating pre-ART care with post-test counseling services should be considered [61,62] as VCT and ART sites were co-located in most cases in Vietnam.

It is also vital to establish the mechanisms to assess and address the needs of PLHIV on ART over the long term. These PLHIV include those who are mobile across provinces and those who have mental health problems, cardio-vascular diseases, cancers and viral hepatitis B and C [5,6].

4) Linkages across different services, administrative boundaries and settings

Integration of HIV testing and counseling into TB and antenatal care services is progressing in districts offering VCT/ART through donor-funded projects. In districts without VCT/ART, no mechanisms appeared to be in place to facilitate access to HIV testing and counseling and to support retention in care.

One option for consideration is to form district clusters [57]. In addition, districts without VCT/ART should explore new approaches to introduce HIV testing and counseling for MARPs, TB cases and pregnant women. Such approaches might include point-of-care HIV diagnosis with rapid-test based algorithms and community outreach testing and counseling [55,56].

The introduction of ART in administrative detention centers has led to the referral system development between these centers and hospitals in the community. It has also triggered the potential of strengthening the basic health care in closed settings including prisons into the coming time.

5) Linkages across different levels of health services

The existing patient flows for TB/HIV, PMTCT and HIV treatment failure management require robust referral systems across the different levels of health services. However, limited information was available to assess the functioning of the referral processes. A simple monitoring system should be developed to assess retention including drop-out and delay during the referral processes.

Administrative boundaries and the hierarchy of authority appear to hinder effective referral services. People-centered health care has been promoted in Asian countries including Vietnam [63]. This approach could help overcome some of the challenges to patient referral.

6) Community response and its linkage with health services

Extensive peer educator schemes established by local health services can be regarded as a major breakthrough in reaching and serving hard-to-reach populations and to ensure adherence to HIV treatment. These schemes are particularly critical in Vietnam where the existence of non-government organizations is limited.

However, a substantial portion of MARPs seems to be still hidden resulting in limited and delayed access to HIV services. Considerations should be given to expanding alternative approaches such as the mobilization of pharmacies and street vendors [54]. Also the capacity of existing peer educators should be maximized. For example, approaches to be considered include promoting synergy between the two schemes of peer educators for prevention and care [64]. Strategies to further decentralize HIV testing and counseling and care and treatment services should be explored. These strategies should include mobile services in the areas where a substantive number of PLHIV have limited physical access to services. These efforts would improve HIV case detection and retention in care and contribute to better outcomes of multiple HIV health services including targeted prevention, TB/HIV, PMTCT and MMT.

### Utility of the COPC framework

This systematic assessment of HIV health service delivery using the COPC framework identified underlying system-related strengths and constraints which affect the performance of multiple HIV health services, particularly HIV case detection and retention in care. Lessons learned from this review could contribute to the optimization of service delivery and the adaptation of Treatment 2.0 to Vietnam’s specific situation [8,9].

While this review focused on HIV health service delivery, the COPC framework could also serve to systematically assess the interfaces between HIV and other health service delivery systems. This is particularly important in the context of the global health agenda to shift from disease control programs to health system strengthening. Such interface could include mechanisms for coordinating and linking different health services, cross-fertilization of management of various chronic illnesses [58], management of patient referral systems, and community mobilization and outreach.

### Limitations

This review is a first attempt to capture the wide range of HIV service delivery processes by using the COPC framework. However, not every element of HIV health services has been addressed. For instance, services for children born to HIV positive mothers have not been included in this analysis. Another limitation of this study is that it includes the analysis of unpublished documents. However, some of the coauthors were involved in the national program from its inception and played important roles in policy development and service delivery. With this knowledge and experience, the authors were able to critically discuss and synthesize each issue presented in the paper.

## Conclusions

This study identified the system-related strengths and constraints of HIV health service delivery in maximizing HIV case detection and retention in care in Vietnam. District-based service delivery models have been developed especially in high burden provinces. Multiple service elements appeared to be connected well in districts offering VCT/ART where donor funded projects were operating. Extensive involvement of PLHIV and MARPs in prevention and care lowered barriers for the marginalized populations to access services. However, service elements tended to be disconnected in districts that were not supported by donor funded projects particularly in middle and low burden provinces. In addition, no adequate service delivery model has been established for MARPs and PLHIV in mountainous remote areas. Based on this review, the national HIV program has initiated pilot projects to address the constraints identified. Furthermore, it plans to strengthen provincial programming by re-defining various service linkages and accelerate the transition from project-based approach to integrated service delivery in line with the Treatment 2.0 initiative. Similar reviews should be considered by the national HIV programs of other countries to optimize respective HIV service delivery models.

## Abbreviations

ART: Antiretroviral treatment;CHBC: Community- and home-based care;COPC: Continuum of prevention and care;HTC: HIV testing and counseling;IDUs: Injecting drug users;MSM: Men who have sex with men;MARPs: Most-at-risk populations;MCH: Maternal and child health;MMT: Methadone maintenance therapy;PLHIV: People living with HIV;PMTCT: Prevention of mother-to-child transmission;SWs: Sex workers;TB: Tuberculosis;VAAC: Vietnam Authority of HIV/AIDS Control;VCT: Voluntary counseling and testing

## Competing interests

The authors declare that they have no competing interests except that KCP has received remuneration from World Health Organization Vietnam to prepare a preliminary draft on the overview of HIV health services in Vietnam.

## Authors’ contributions

MF and KCP designed the study, coordinated the collection, analysis and interpretation of data and drafted the manuscript. MK, NTMT and DJ participated in design of the study, analysis and interpretation of data and drafting of the manuscript. KG contributed to data collection and revisions of the entire manuscript. DTN, BDD, NVK, CTTT, NTL contributed to conceptualization of the study, participated in data collection and provided inputs to the manuscript. MJ contributed to refinement of methods, interpretation of data and revisions of the manuscript. All authors read and approved the final manuscript.

## Authors’ information

MF and KG were with World Health Organization Vietnam and FHI 360 Vietnam respectively during the study period.

## Pre-publication history

The pre-publication history for this paper can be accessed here:

http://www.biomedcentral.com/1472-6963/12/483/prepub
